# Automated quantification of COVID-19 pneumonia severity in chest CT using histogram-based multi-level thresholding segmentation

**DOI:** 10.1186/s43055-021-00602-1

**Published:** 2021-12-09

**Authors:** Hazem Abuzeid Yousef, Ehab Mansour Mohmad Moussa, Mohamed Zidan Mohamed Abdel-Razek, Maha Mohamed Said Ahmed El-Kholy, Lamiaa Hasan Shaaban Hasan, Alaa El-Din Abdel-Moneim El-Sayed, Medhat Araby Khalil Saleh, Mohamed Karim Mahmoud Omar

**Affiliations:** 1 Radiodiagnosis Department, Faculty of Medicine, Assiut University, Assiut, Egypt; 2 Department of Chest Diseases, Faculty of Medicine, Assiut University, Assiut, Egypt; 3 Department of Internal Medicine, Faculty of Medicine, Assiut University, Assiut, Egypt; 4 Department of Public Health and Community Medicine, Faculty of Medicine, Assiut University, Assiut, Egypt

**Keywords:** COVID-19, Pneumonia, Computed tomography (CT), Histogram-based, Quantitative analysis, Severity scoring

## Abstract

**Background:**

Chest computed tomography (CT) has proven its critical importance in detection, grading, and follow-up of lung affection in COVID-19 pneumonia. There is a close relationship between clinical severity and the extent of lung CT findings in this potentially fatal disease. The extent of lung lesions in CT is an important indicator of risk stratification in COVID-19 pneumonia patients. This study aims to explore automated histogram-based quantification of lung affection in COVID-19 pneumonia in volumetric computed tomography (CT) images in comparison to conventional semi-quantitative severity scoring. This retrospective study enrolled 153 patients with proven COVID-19 pneumonia. Based on the severity of clinical presentation, the patients were divided into three groups: mild, moderate and severe. Based upon the need for oxygenation support, two groups were identified as follows: common group that incorporated mild and moderate severity patients who did not need intubation, and severe illness group that included patients who were intubated. An automated multi-level thresholding histogram-based quantitative analysis technique was used for evaluation of lung affection in CT scans together with the conventional semi-quantitative severity scoring performed by two expert radiologists. The quantitative assessment included volumes, percentages and densities of ground-glass opacities (GGOs) and consolidation in both lungs. The results of the two evaluation methods were compared, and the quantification metrics were correlated.

**Results:**

The Spearman’s correlation coefficient between the semi-quantitative severity scoring and automated quantification methods was 0.934 (*p* < 0.0001).

**Conclusions:**

The automated histogram-based quantification of COVID-19 pneumonia shows good correlation with conventional severity scoring. The quantitative imaging metrics show high correlation with the clinical severity of the disease.

## Background

The 2019 novel coronavirus (SARS-CoV-2) disease (COVID-19) began in early December of 2019 and rapidly spread across the globe to become a pandemic [1–3]. COVID-19 pandemic presented an unprecedented challenge to the global healthcare system. Many COVID-19 patients develop pneumonia that may progress rapidly into severe acute respiratory distress syndrome (ARDS) with grave prognosis and high mortality [4, 5].

Chest computed tomography (CT) has proven its sensitivity for early diagnosis of COVID-19 pneumonia [6]. CT has proven critical importance in detection, grading, and follow-up of lung affection in this potentially fatal disease. The important role of the radiologists in COVID-19 infection is to provide early diagnosis, distinguish the disease from other conditions with similar CT findings and determine disease severity [7, 8].

Positive CT findings may predate positivity on real-time reverse transcription-polymerase chain reaction (RT-PCR) testing, which is the gold standard for diagnosis, and it could detect minor lung lesions at an early stage of the disease. The diagnosis and treatment protocols have been modified in the context of this pandemic to include clinically suspected patients with characteristic CT features of COVID-19 pneumonia but negative RT-PCR results [9–12].

Typical CT findings in COVID-19 pneumonia include initially, bilateral ground-glass opacities (GGOs) located peripherally, followed later by consolidation, with multi-lobe affection of both lungs in most cases. Crazy paving, reticulation and reversed halo sign are encountered with progression of the disease [13–17]. COVID-19 tends to cause severe changes on CT that reflect the widespread aggressive lung injury characteristic of this disease [18].

Lung affection patterns including the number, size, and density of lesions, as well as the overall extent of diseased lung, are all indicators of lung damage and remaining lung reserve. Identifying objective prognostic parameters in CT images of COVID-19 patients may allow better management decisions that hopefully lead to improved clinical outcome [19].

The great sensitivity of chest CT for diagnosing COVID-19 pneumonia poses a significantly increasing workload on radiologists to visually identify and evaluate the extent of COVID-19 affection from thin-thickness CT images, which becomes more challenging if follow-up CT imaging is needed [20, 21].

Previous studies have shown a close relationship between clinical and lung CT severity in COVID-19 pneumonia [14, 22, 23]. The extent of lung lesions in CT is an important indicator of risk stratification in COVID-19 patients [19].

Quantitative CT which is already widely used for assessment of diffuse lung diseases, especially in interstitial disease, is an objective tool for detection of image features that are not visually recognizable by the radiologists [24, 25].

The internationally adopted COVID-19 Reporting and Data System (CO-RADS), recommended by the Radiological Society of North America and other radiological societies [26, 27], uses a scoring system from 0 to 5 to classify lung involvement in CT images from very unlikely to very likely, respectively. The CO-RADS has shown a very good performance for predicting the likelihood of COVID-19 infection with substantial inter-observer agreement [28].

Most of the imaging studies since the outbreak of COVID-19 have focused on lung CT findings, with only few studies concerning the quantitative analysis of these findings [29].

Unfortunately, this role has been reluctantly integrated into the routine radiological practice because the radiologist's semi-quantitative visual assessment is subjective, time consuming and lacking inter-observer consistency. A semi-quantitative visual severity scoring (SS) system for lung affection has been proposed [30] for assessment of lung affection in SARS, and for evaluation of ARDS [31].

The scoring of disease severity is performed in each of the five lung lobes on a scale from 0 to 5, with 0 indicating no involvement, 1 indicating less than 5% involvement, 2 indicating 5–25% involvement, 3 indicating 26–49% involvement, 4 indicating 50–75% involvement, and 5 indicating more than 75% involvement. The sum of individual lobar scores represents the total SS that ranges from 0 (no involvement) to 25 (maximum involvement). This semi-quantitative lobar-based visual scoring system has been adopted in the assessment of the severity of COVID-19 lung affection [15, 32, 33].

Quantitative CT analysis is superior to semi-quantitative visual SS in assessment of the severity of COVID-19 infection. Computerized quantitative segmentation methods could provide objective assessment of the percentage of the diseased part of the lung containing GGOs and consolidation to determine the disease burden [34, 35].

The lung volumes measured by CT are well correlated with pulmonary function test results such as total lung capacities and forced vital capacities [36]. Quantitative indices have higher reproducibility than visual scoring and are significantly correlated with lung function and clinical parameters [37].

Still there is no consensus regarding the grading of severity of lung affection in CT images of COVID-19 pneumonia. Simple subjective descriptive terms are cordially used by most radiologists for determination of the severity of lung affection such as mild, moderate, severe or critical.

This study aims to explore an automated histogram-based quantitative CT method together with the conventional semi-quantitative visual severity scoring for assessment of COVID-19 pneumonia and to correlate the quantitative metrics with clinical and laboratory findings in COVID-19 pneumonia patients.

## Methods

### Patients

This is a single-center retrospective study that included 153 patients with COVID-19 infection (79 male, 74 female; age range from 19 to 78 years, mean age, 53 ± 14 years) who were presented to the emergency ward of Assiut University Hospitals during the period from March to July 2020. COVID-19 was diagnosed based on a positive result of RT-PCR assay on pharyngeal swab specimens. Chest CT scanning was performed within 3–6 days after the onset of symptoms. The clinical and laboratory data of these patients were collected. The patient characteristics are summarized in Table 1.

**Table 1:** Clinical and laboratory characteristics of patients

*n* = 153	*n*/Mean (SD)
Age (year)	53 (14)
Female:male	74:79
Onset of symptoms (day)	4.3
Symptoms	
Sore throat	112
Fever	141
Malaise	151
Headache	141
Nasal congestion	106
Dyspnea	118
Dry cough	126
Loss of the taste and/or smell	132
Muscle and or joint pain	146
Nausea or vomiting	63
Diarrhea	57
Laboratory results	
Lymphocytes (%)	17.6 (4.5)
C. reactive protein (mg/dL)	106 (48)
Lactate dehydrogenase (u/L)	448 (231)
Ferritin (ng/mL)	571 (368)
D. Dimer (mg/mL)	2.1
Comorbid diseases	
Hypertension	84
Diabetes	78
Chronic kidney disease	31
Chronic liver disease	26
Smoking	46

Based on the clinical presentation, the patients were divided into three groups: mild illness: individuals who have any of the various signs and symptoms of covid-19 (e.g., fever, cough, sore throat, malaise, headache, muscle pain, nausea, vomiting, diarrhea, loss of taste and smell) but who do not have shortness of breath, dyspnea, or abnormal chest imaging findings; moderate illness: individuals who show evidence of lower respiratory tract disease during clinical assessment or imaging and who have an oxygen saturation (SpO_2_) ≥ 94% on room air; severe illness: individuals who have spO_2_ < 94% on room air, a ratio of arterial partial pressure of oxygen to fraction of inspired oxygen (PaO2/FiO2) < 300 mm hg, respiratory frequency > 30 breaths/min, or lung infiltrates > 50% [38].

### CT examination

Whole lung volumetric CT scanning was performed using a 16-row multi-detector CT scanner (BrightSpeed 16; General Electric Healthcare, Milwaukee, USA). Scanning was performed from lung apices to the diaphragm during a single breath-hold at deep inspiration, using the following parameters: tube voltage 120 kVp; automatic tube-current modulation; gantry rotation speed of 0.5 s; and beam collimation of 16 × 0.625 mm. Thin-section CT data were reconstructed at 0.625 mm thickness using standard filtered back-projection algorithm; iterative reconstruction algorithms were not used.

### Infection control

The CT technologist and the attending nurse routinely wore personal protective equipment (PPE) while handling patients in the CT suite. The PPE included transparent face-shield, a surgical cap, a surgical mask, gloves, a fluid-resistant gown, and shoe covers. Decontamination of the CT machine was performed routinely after finishing CT scanning according to the infection control guidelines. Disposable sheets were used to cover the CT imaging table. The patients wore disposable masks and head caps before entering the CT examination room.

### Semi-quantitative visual severity scoring assessment

The high-resolution computed tomography (HRCT) images were independently reviewed by two expert radiologists on a picture archiving and communication system (PACS) computer workstation at window setting for lung parenchyma (center, − 600 HU; width, 1600 HU). A lobar-based visual SS was independently identified by two expert radiologists, and the scores were then averaged to determine the mean total SS of COVID-19 lung affection. The scoring system considered the overall extent of parenchymal abnormalities, including the GGOs and consolidation (Co), using the definitions of the Fleischner Society glossary of terms for thoracic imaging [39]. Any co-existing reticular pattern (inter-lobular, intra-lobular and/or peri-bronchial thickening) or other types of opacities (crazy paving or reversed halo), or pleural effusion were also documented alongside the severity scoring.

### Post-processing and quantitative CT measures

A commercially available computer workstation, Synapse 3D version 3.5 (Fujifilm Medical Systems, Tokyo, Japan), was used for quantitative analysis of CT images. The digital imaging and communications in medicine (DICOM) data of the CT scans of all patients were transferred to the workstation from the scanner. Whole lung extraction was automatically performed by eliminating the thoracic wall, mediastinum, large vessels, and tracheo-bronchial airway down to tertiary bronchi. The lung extraction process (according to vendor's data) uses both Hounsfield thresholding and anatomical knowledge-based algorithms.

An additional COVID-19 analysis dataset was added to the Synapse 3D workstation for analysis of COVID-19 pneumonia in CT images. The dataset consists of 4 groups of density ranges:

- From − 1024 to − 950 HU (red), representing emphysema (low-attenuation areas, LAA).
- From − 949 to − 750 HU (yellow), corresponds to healthy lung tissue.
- From − 749 to − 300 HU (blue), it represents the lung parts which are more dense than healthy lung (high-attenuation areas, HAA) and can be used to quantify ground-glass opacities
- From − 299 to + 40 HU (violet), this group corresponds to areas with further increase in density, including the semi-consolidation and consolidation.

For density-based quantitative analysis, 1.5 mm high-resolution slices were reconstructed at sharp kernel settings. The lung analysis software of the workstation automatically generates the histogram of distribution of the density of each voxel within the lung and calculates the mean of distribution. In quantitative lung analysis, the following metrics were automatically extracted from the lung density histogram:

- The volume of each lung and the total volume of both lungs (TLV) in cubic centimeter (cc)
- The mean density of each lung and the mean density of both lungs (MLD) in HU
- The volume and percentage of GGOs (_vol_GGO and _%_GGO, respectively) and the volume and percentage of consolidation (_vol_Co and _%_Co, respectively) in both lungs. The total volume of diseased lung was manually calculated as the sum of _vol_GGO and _vol_Co, and the total percentage of the diseased lung or total lesion load (TLL), representing the disease burden, was calculated as the sum of _%_GGO and _%_Co.
- The volumes and percentages of normal and hyperinflated parts of both lungs

These numerical data were expressed as mean ± standard deviation (SD).

### Statistical analysis

Statistical analysis was performed using MedCalc Statistical Software version 20 (MedCalc Software Ltd, Ostend, Belgium). Inter-observer agreement between the two radiologists who performed semi-quantitative analysis of CT images of patients of the study was evaluated using kappa test. Pearson correlation coefficients were calculated between the visual severity scoring and the automated quantitative measures; between the total disease burden calculated by quantitative CT analysis and the MLD; and finally between the _%_GGO and MLD. *p* value of less than 0.05 was considered statistically significant.

## Results

The clinical and laboratory data of the patients included in the study are shown in Table 1. The CT features which were observed in patients of the study were as follows (Table 2): most of the patients presented with ground-glass opacities (149 patients, 97.3%); alveolar consolidation was encountered in 67 patients (43.7%); crazy-paving pattern in 11 patients (16.8%); reversed halo sign in 4 patients (6.1%); and pleural effusion in 12 (18.2%). Both lungs were involved in most of the patients (145 patients, 94.8%); with multi-lobar affection (146 patients, 95.4%). The changes affected mainly the lower lobes in 103 patients (67.3%).

**Table 2:** Depicted lung abnormalities in chest CT

Finding	*n* (%)
Lung abnormality	
GGOs only	86 (56.2%)
Consolidation only	4 (2.6%)
GGOs and consolidation	63 (41.1%)
Crazy paving	11 (7.1%)
Reversed halo sign	4 (2.6%)
Pleural effusion	12 (7.8%)
Laterality	
Unilateral	8 (5.2%)
Bilateral	145 (94.8%)
Lobar affection	
Single lobe	7 (4.6%)
Multiple lobes	146 (95.4%)

The calculated quantitative metrics including volumes, percentages and densities are summarized in Table 3.

**Table 3:** Quantitative data of lung lesions in chest CT of COVID-19 pneumonia patients

	Group1 (common) *n* = 104 Mean (SD)	Range	Group 2 (severe) *n* = 49 Mean (SD)	Range
Total lung volume	3312.8 (920.8)	1719 to 4982	2131.8 (462.3)	1454 to 3997
Lung lesion volume (cc)				
_vol_GGOs	1027.7 (298.5)	441 to 1863	1236.9 (243.1)	720 to 1894
_vol_Consolidation	63.8 (21.4)	48 to 142	82.7 (24.6)	76 to 203
Total	1257.3 (154.9)	503 to 2021	1331.5 (117)	783 to 2123
Lung lesion burden (%)				
_%_GGOs	32.6 (10.3)	9.7 to 48.5	58.6 (7.1)	48.7 to 73.2
_%_Consolidation	1.1 (0.4)	0.5 to 2.1	1.9 (0.7)	1.2 to 3.4
Total	33.9 (11.2)	10.3 to 49.6	60.7 (7.4)	51.3 to 73.1
Mean lung density	− 768.6 (36.9)	− 660 to − 854	− 685.9 (34.1)	− 604 to − 771
Lesion mean attenuation				
GGOs	− 543.5 (60.8)	− 341 to − 687	− 496.7 (51.3)	− 327 to − 628
Consolidation	− 11.7 (24.8)	− 29 to 47	− 6.4 (48.9)	− 3 to 68

The inter-observer agreement of severity scoring between both radiologists who performed semi-quantitative evaluation of lung lesions in this study (Weighted Kappa) is 0.7984 ± 0.0140, with 95% confidence interval of 0.7708–0.8261. The concordance correlation coefficient between the two radiologists is 0.9615 with 95% confidence interval of 0.9474–0.9719.

**Fig. 1:**
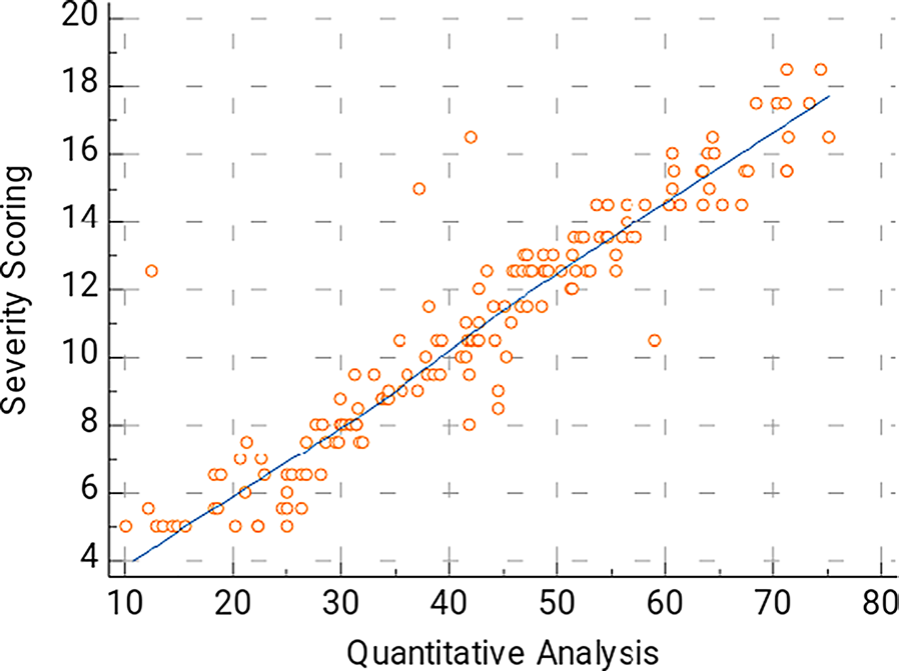
The correlation between the disease burden (% compromised lung) calculated by automated quantification and semi-quantitative severity scoring. This is a strong positive correlation; *r* = 0.934, 95%CI for *r* = 0.9092–0.9512

**Fig. 2:**
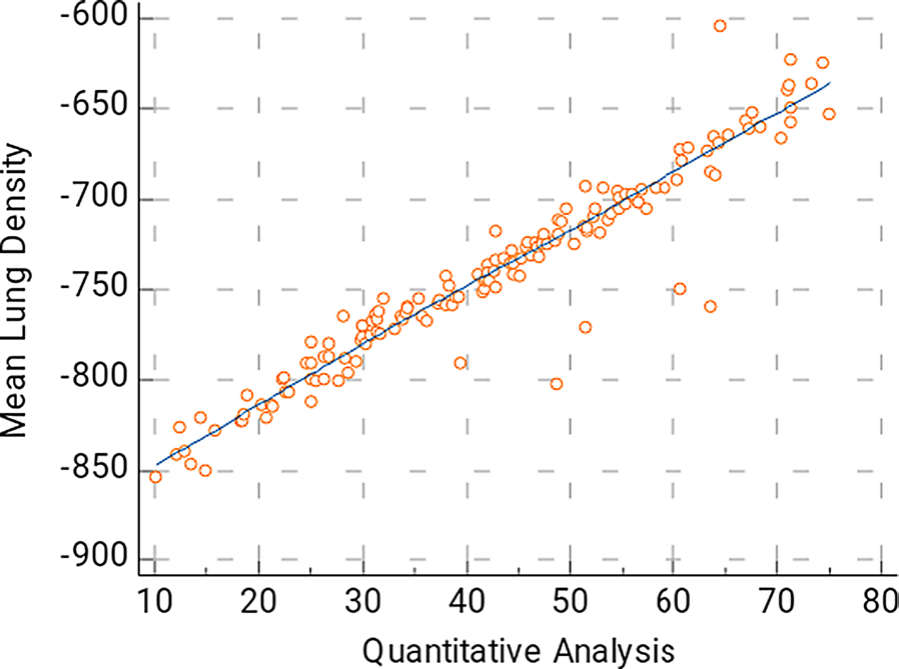
The correlation of between the disease burden (% compromised lung) calculated by automated quantification and MLD. This is a strong positive correlation; *r* = 0.9544, the 95% CI for *r* = 0.9376–0.9667

**Fig. 3:**
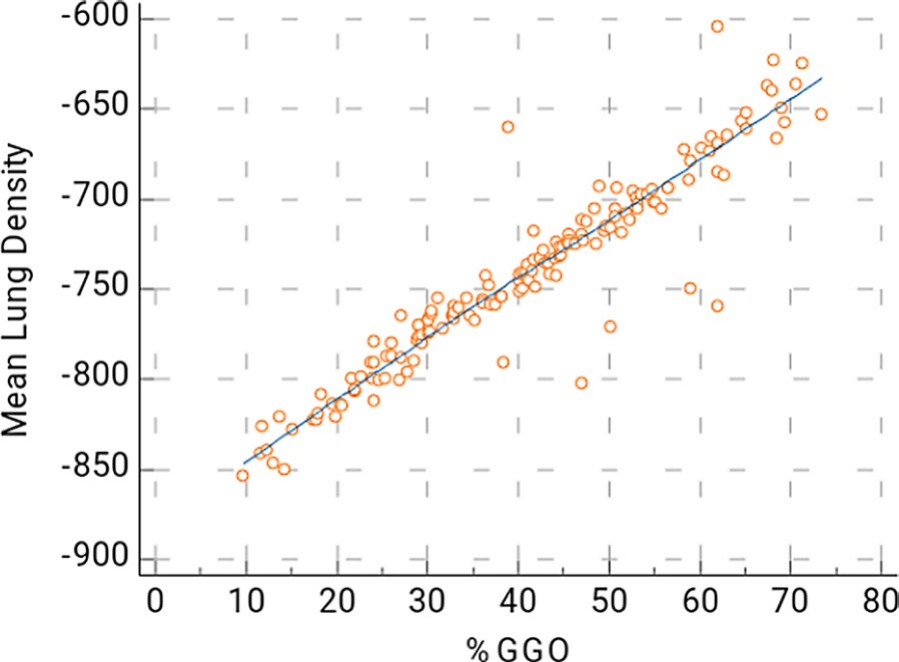
The correlation of between the MLD and _%_GGO. This is a strong positive correlation; *r* = 0.9429, the 95% confidence interval for *r* = 0.9222–0.9582

In this study, Spearman’s correlation coefficient shows strong positive correlation between the semi-quantitative CT severity scoring and the automated quantification method in all patients is 0.934 (*p* < 0.0001), with confidence interval (CI) for r between 0.9092 and 0.9512 (Fig. 1). There is also strong positive correlation coefficient between the quantitative analysis and the MLD is (*r* = 0.9544, 95% CI 0.9376–0.9667, *p* < 0.0001) (Fig. 2). There is also strong positive correlation between the %GGO and MLD (*r* = 0.9429, 95% CI 0.9222–0.9582, *p* < 0.0001) (Fig. 3).

**Fig. 4:**
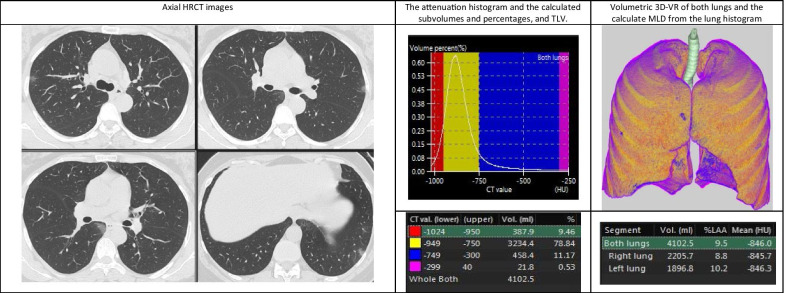
Quantitative CT analysis of COVID-19 pneumonia in 32-year-old woman suffering from mild symptoms that required hospitalization with open mask oxygenation. Axial HRCT images show multiple small sub-pleural GGOs scattered in the upper and lower lobes of both lungs. Volumetric 3D-VR shows multiple small blue areas affecting both lungs representing the diseased lung. The patient had 11.7% disease burden

**Fig. 5:**
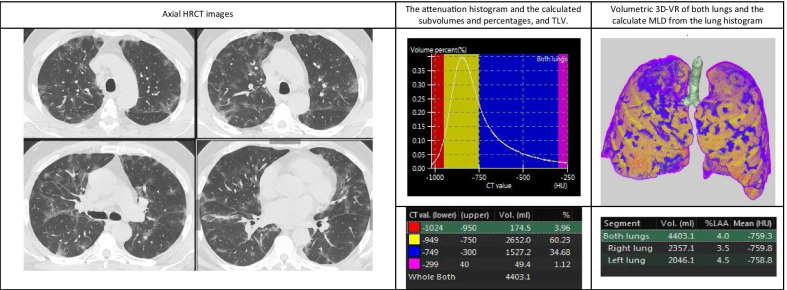
Quantitative CT analysis of COVID-19 pneumonia in 53-year-old man with moderate symptoms that required intermediate ICU admission with high-flow oxygenation support. Axial HRCT images show multiple bilateral relatively large sub-pleural GGOs affecting all lobes. The blue and pink areas in volumetric 3D-VR reflect GGOs and consolidation. The patient had 35.8% disease burden

**Fig. 6:**
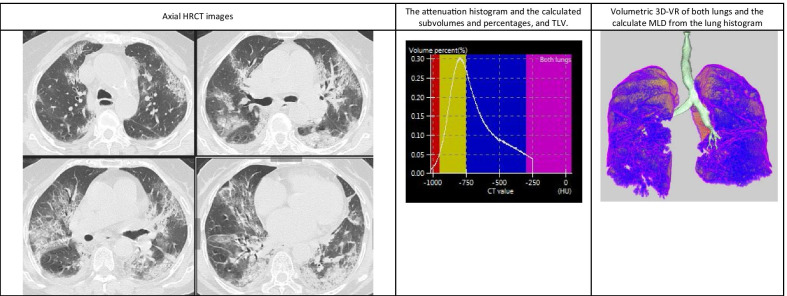
Quantitative CT analysis of COVID-19 pneumonia in 64-year-old man suffering from severe symptoms that required ICU admission with high-flow oxygenation using nasal cannula. Axial HRCT images show multiple large sub-pleural GGOs affecting all lobes of both lungs with few consolidations. Volumetric 3D-VR shows extensive bilateral blue and pink areas representing GGOs and consolidation. The patient had 54.3% disease burden

Demonstrative data of the quantitative findings in three cases of the study are shown in Figs. 4, 5 and 6 including axial HRCT images, 3D volume-rendered images of both lungs, density histograms and calculated metrics.

## Discussion

The key for containment of COVID-19 pandemic is early detection and early isolation [40]. CT plays an important role in COVID-19 diagnosis, monitoring, severity stratification, and evaluation of treatment response [6, 41, 42]. The overall CT picture of COVID-19 pneumonia is based on the severity of lung abnormalities and its distribution. The severity of COVID-19 pneumonia should be objectively stratified on the basis of quantitative data. The severity of lung affection is a critical metric in treatment and prognosis of COVID-19 patients [19, 25]. Severe abnormalities in lung CT at an early stage are suggestive of poor prognosis [40].

Fast, accurate, and reproducible quantitative analytical tools are especially needed for assessment of COVID-19 pneumonia in CT images because, in addition to being multi-focal, lung lesions often show rapid progression and change of its pattern [33].

Semi-quantitative visual assessment of COVID-19 lesions is impractical in clinical routine because it is time-consuming, lacks reproducibility and suffers inter-observer and even intra-observer variations. The objective assessment of the disease burden expressed as the percentage of the affected lung relative to the total lung volume is a sensitive and specific metric for estimation of disease progression and treatment response [15, 34].

Computer-aided diagnosis (CAD) has become an important auxiliary diagnostic tool. The automated segmentation of lung lesions from volumetric 3D images allows calculation of the total burden of COVID-19 pneumonia as a percentage subvolume of the total lung volume. Quantitative methods for determination of the severity of lung affection in CT images of covid-19 patients could improve the diagnostic efficiency and mitigate the workload of radiologists, allowing more timely and appropriate treatment decisions for COVID-19 patients. Quantitative CT analysis represents a reproducible assessment that allows fast, reliable and potentially predictive tool for assessing disease progression and response to treatment in COVID-19 pneumonia [43].

Another advantage of computer-assisted quantification methods for determination of COVID-19 disease burden is the reproducibility of the technique, allowing for more accurate comparison of data among different centers. Different patterns of lung abnormalities in COVID-19 pneumonia change the lung attenuation values and affect its histogram. The histogram-based image analysis is the most basic and least computation demanding segmentation technique, implemented in most PACS workstations being simple, fast and reproducible [44].

Image thresholding is one of the most commonly used image segmentation technique that segment images depending upon the grayscale values within the image histogram. Multi-level thresholding CT densitometry techniques rely upon choosing multiple cutoff values in the analysis of the frequency distribution of the lung attenuation values in the histogram. The pixels within an image are divided into multiple classes according to multiple grayscale thresholds [45–47].

With the rapid increase of the infected population in the COVID-19 pandemic, semi-quantitative conventional CT scoring is challenging and impractical in the overloaded Radiology service.

In this study, the density-based multi-level thresholding technique was utilized for quantification of COVID-19 lung affection in high-resolution thin-cuts volumetric whole-lung CT images. The findings of this study show that the extent of COVID-19 lesions visually scored by radiologists from HRCT images does significantly correlate with the volumetric measurements obtained by the quantitative computer-assisted histogram-based automatic method.

The data obtained in this study show that both semi-quantitative visual severity scoring and quantitative CT performed nearly equally, and their parameters correlated well with the clinical data of patients. Our findings indicate that the severity of COVID-19 pneumonia could be accurately stratified on the basis of objective CAD-based quantitative data of disease burden in CT images.

This is consistent with the results of Li et al. [48] who described a high consistency between COVID-19 severity scoring assessed by visual semi-quantitative CT analysis with the clinical classification of COVID-19 pneumonia. On the other hand, Colombi et al. [24] reported a good correlation between the well-aerated lung volume and the patient's clinical outcome in COVID-19 pneumonia.

Lanza et al. [49] used density-based quantitative lung analysis to predict clinical outcome in COVID-19 pneumonia regarding the need for respiratory support and the risk of in-hospital mortality. They reported that the compromised lung volume was the most accurate metric in this regard.

Bressem et al. [50] used density-based classification to detect correlation between high-density lung volume (diseased lung) and the severity of COVID-19 pneumonia requiring intensive care unit (ICU) admission and assisted ventilation.

In a recent study, Salvatore et al. [51] used a computer-assisted density-based quantitative technique for automatic segmentation of CT images to calculate the volumes of GGOs, consolidation and the residual healthy lung in COVID-19 pneumonia. Their findings have shown that the results of these quantitative methods are good predictors of COVID-19 patient outcome.

In another recent study, Romanov et al. [52] used histogram-based analysis and HU thresholding to automatically extract CT imaging biomarkers in atypical pneumonia caused by COVID-19 and influenza viruses. The authors reported that the derived imaging biomarkers correlate with the clinical severity scale and the inflammatory laboratory markers.

Semi-quantitative scoring of the extent of COVID-19 pneumonia based upon visual assessment has been shown to correlate with the duration of infection [15, 52] as well as with the disease severity [35, 49].

In ARDS, a ratio less than 40% between well-aerated lung volume and the total lung capacity was reported to be associated with a higher mortality risk [43].

Colombi et al. [24] used lung attenuation thresholds to quantify well-aerated lung volume in admittance CT images and stated that it can be used to predict the risk of adverse outcome in COVID-19 patients. CT score of the diseased lung has been reported as a risk factor for mortality in ARDS [53].

Patients with COVID-19 lung affection frequently develop ARDS [54, 55] which is the primary cause of death in COVID-19 pneumonia, especially in old aged patients with comorbidities [25].

In all cases of this study regardless its clinical or radiological severity, in presence of GGOs, the automatically extracted whole lung attenuation histograms show blunted peak that is shifted to the right compared with the normal lung, while, in presence of alveolar consolidation, the histograms show a high sharp peak that is shifted more to the right compared to ground-glass opacities and normal lung. These findings are consistent with those described by Sumikawa et al. [44].

## Conclusions

The results of this study show that the automated histogram-based quantification of COVID-19 disease burden is a rapid, reliable and reproducible method for objective estimation of lung affection in CT images. There is good correlation between the conventional semi-quantitative CT severity scoring and automated quantitative analysis methods. The automated quantitative methods are especially important in situations like the current COVID-19 pandemic in which radiology departments are overloaded with cases.

Some limitations do exist in this study. First and foremost, the lack of longitudinal assessment of disease progression of patients enrolled in the study. The study also included a relatively small number of patients.

## Data Availability

The anonymized datasets used and analyzed during the current study are available on reasonable request from the corresponding author. Ethical approvals were not obtained to allow public sharing of raw imaging data from patients sharing in the study.

## Abbreviations

SARS-CoV-2: Severe acute respiratory syndrome coronavirus 2
COVID-19: The novel coronavirus, coronavirus disease 2019
ARDS: Acute respiratory distress syndrome
CT: Computed tomography
RT-PCR: Real-time reverse transcription-polymerase chain reaction
GGOs: Ground-glass opacities
Co: Consolidation
CO-RADS: COVID-19 reporting and data system
SS: Severity score
SpO_2_: Peripheral oxygen saturation
PaO_2_: Arterial oxygen partial pressure
FiO_2_: Fractional inspired oxygen
PPE: Personal protective equipment
HRCT: High-resolution computed tomography
PACS: Picture archiving and communication systems
DICOM: Digital imaging and communication in medicine
HU: Hounsfield unit
HAA: High attenuation areas
TLV: Total lung volume
MLD: Mean lung density
_vol_GGOs: Volume of ground-glass opacities
_%_GGOs: Percentage of ground-glass opacities
_vol_Co: Volume of consolidation
_%_Co: Percentage of consolidation
TLL: Total lesion load
SD: Standard deviation
3D: Three-dimensional
CAD: Computer-aided diagnosis
ICU: Intensive care unit
CI: Confidence interval

## References

[1] Munster VJ, Koopmans M, van Doremalen N, van Riel D, de Wit E (2020). A novel coronavirus emerging in China—key questions for impact assessment. New Engl J Med.

[2] Wang C, Horby PW, Hayden FG, Gao GF (2020). A novel coronavirus outbreak of global health concern. Lancet.

[3] Cucinotta D, Vanelli M (2020). WHO declares COVID-19 a pandemic. Acta Biomed.

[4] Guan W, Ni Z, Hu Y, Liang W, Ou C, He J (2020). Clinical characteristics of coronavirus disease 2019 in China. N Engl J Med.

[5] Huang C, Wang Y, Li X, Ren L, Zhao J, Hu Y (2020). Clinical features of patients infected with 2019 novel coronavirus in Wuhan, China. Lancet.

[6] Ai T, Yang ZL, Hou HY (2020). Correlation of chest CT and RT-PCR testing in coronavirus disease 2019 (COVID-19) in China: a report of 1014 cases. Radiology.

[7] Kanne JP, Little BP, Chung JH, Elicker BM, Ketai LH (2020). Essentials for radiologists on COVID-19: an update-radiology scientific expert panel. Radiology.

[8] Zu ZY, Jiang MD, Xu PP, Chen W, Ni QQ, Lu GM (2020). Coronavirus disease 2019 (COVID-19): a perspective from China. Radiology.

[9] Xie X, Zhong Z, Zhao W, Zheng C, Wang F, Liu J (2020). Chest CT for typical 2019-nCoV pneumonia: relationship to negative RT-PCR testing. Radiology.

[10] Liu J, Yu H, Zhang S (2020). The indispensable role of chest CT in the detection of coronavirus disease 2019 (COVID-19). Eur J Nucl Med Mol Imaging.

[11] Dai H, Zhang X, Xia J, Zhang T, Shang Y, Huang R (2020). High-resolution chest CT features and clinical characteristics of patients infected with COVID-19 in Jiangsu, China. Int J Infect Dis.

[12] Huang P, Liu T, Huang L, Liu H, Lei M, Xu W (2020). Use of chest CT in combination with negative RT-PCR assay for the 2019 novel coronavirus but high clinical suspicion. Radiology.

[13] Salehi S, Abedi A, Balakrishnan S, Gholamrezanezhad A (2020). Coronavirus disease 2019 (COVID-19): a systematic review of imaging findings in 919 patients. AJR Am J Roentgenol.

[14] Zhao W, Zhong Z, Xie X, Yu Q, Liu J (2020). Relation between chest CT findings and clinical conditions of coronavirus disease (COVID-19) pneumonia: a multicenter study. AJR Am J Roentgenol.

[15] Pan F, Ye T, Sun P (2020). Time course of lung changes on chest CT during recovery from 2019 novel coronavirus (COVID-19) pneumonia. Radiology.

[16] Shi H, Han X, Jiang N, Cao Y, Alwalid O, Gu J, Fan Y, Zheng C (2020). Radiological findings from 81 patients with COVID-19 pneumonia in Wuhan, China: a descriptive study. Lancet Infect Dis.

[17] Bernheim A, Mei X, Huang M (2020). Chest CT findings in coronavirus disease-19 (COVID-19): relationship to duration of infection. Radiology.

[18] Wu J, Wu X, Zeng W, Guo D, Fang Z, Chen L, Huang H, Li C (2020). Chest CT findings in patients with coronavirus disease 2019 and its relationship with clinical features. Investig Radiol.

[19] Zhang K, Liu X, Shen J, Li Z, Sang Y, Wu X (2020). Clinically applicable AI system for accurate diagnosis, quantitative measurements, and prognosis of COVID-19 pneumonia using computed tomography. Cell.

[20] Wu X, Hui H, Niu M, Li L, Wang L, He B, Yang X (2020). Deep learning-based multi-view fusion model for screening 2019 novel coronavirus pneumonia: a multicentre study. Eur J Radiol.

[21] Yang S, Jiang L, Cao Z, Wang L, Cao J, Feng R, Zhang Z, Xue X, Shi Y, Shan F (2020). Deep learning for detecting corona virus disease 2019 (COVID-19) on high-resolution computed tomography: a pilot study. Ann Transl Med.

[22] Yılmaz Demirci N, UğraşDikmen A, Taşçı C, Doğan D, Arslan Y, Öcal N (2021). Relationship between chest computed tomography findings and clinical conditions of coronavirus disease (COVID-19): a multicentre experience. Int J Clin Pract.

[23] Quispe-Cholan A, Anticona-De-La-Cruz Y, Cornejo-Cruz M (2020). Tomographic findings in patients with COVID-19 according to evolution of the disease. Egypt J Radiol Nucl Med.

[24] Colombi D, Bodini FC, Petrini M, Maffi G, Morelli N, Milanese G (2020). Well-aerated lung on admitting chest CT to predict adverse outcome in COVID-19 pneumonia. Radiology.

[25] Suri JS, Agarwal S, Gupta SK, Puvvula A, Biswas M, Saba L (2021). A narrative review on characterization of acute respiratory distress syndrome in COVID-19-infected lungs using artificial intelligence. Comput Biol Med.

[26] Simpson S, Kay FU, Abbara S (2020). Radiological society of North America expert consensus statement on reporting chest CT findings related to COVID-19. Endorsed by the society of thoracic radiology, the American College of Radiology, and RSNA. J Thorac Imaging.

[27] Prokop M, van Everdingen W, van Rees Vellinga T (2020). CO-RADS: a categorical CT assessment scheme for patients with suspected COVID-19: definition and evaluation. Radiology.

[28] Lieveld AWE, Azijli K, Teunissen BP, van Haaften RM, Kootte RS, van den Berk IAH (2021). Chest CT in COVID-19 at the ED: validation of the COVID-19 reporting and data system (CO-RADS) and CT severity score: a prospective, multicenter, observational study. Chest.

[29] Qiu J, Peng S, Yin J, Wang J, Jiang J, Li Z (2021). A radiomics signature to quantitatively analyze COVID-19-infected pulmonary lesions. Interdiscip Sci.

[30] Chang YC, Yu CJ, Chang SC, Galvin JR, Liu HM, Hsiao CH (2005). Pulmonary sequelae in convalescent patients after severe acute respiratory syndrome: evaluation with thin-section CT. Radiology.

[31] Ichikado K, Suga M, Muranaka H (2006). Prediction of prognosis for acute respiratory distress syndrome with thin-section CT: validation in 44 cases. Radiology.

[32] Francone M, Iafrate F, Masci GM, Coco S, Cilia F, Manganaro L (2020). Chest CT score in COVID-19 patients: correlation with disease severity and short-term prognosis. Eur Radiol.

[33] Guan X, Yao L, Tan Y, Shen Z, Zheng H, Zhou H (2021). Quantitative and semi-quantitative CT assessments of lung lesion burden in COVID-19 pneumonia. Sci Rep.

[34] Huang L, Han R, Ai T, Yu P, Han K, Qian T (2020). Serial quantitative chest CT assessment of COVID-19: deep-learning approach. Radiology.

[35] Yang R, Li X, Liu H, Zhen Y, Zhang X, Qiuxia X, Luo Y (2020). Chest CT severity score: an imaging tool for assessing severe COVID-19. Radiology.

[36] Robbie H, Wells AU, Jacob J, Walsh SLF, Nair A, Srikanthan A (2019). Visual and automated CT measurements of lung volume loss in idiopathic pulmonary fibrosis. AJR Am J Roentgenol.

[37] Yin X, Min X, Nan Y, Feng Z, Li B, Cai W (2020). Assessment of the severity of coronavirus disease: quantitative computed tomography parameters versus semiquantitative visual score. Korean J Radiol.

[38] COVID-19 Treatment Guidelines Panel (2021) Coronavirus disease 2019 (COVID-19) treatment guidelines. National Institutes of Health. Available at https://www.covid19treatmentguidelines.nih.gov/. Accessed 15 June 2021 34003615

[39] Hansell DM, Bankier AA, MacMahon H, McLoud TC, Müller NL, Remy J (2008). Fleischner society: glossary of terms for thoracic imaging. Radiology.

[40] Peck KR (2020). Early diagnosis and rapid isolation: response to COVID-19 outbreak in Korea. Clin Microbiol Infect.

[41] Li Y, Xia LM (2020). Coronavirus disease 2019 (COVID-19): role of chest CT in diagnosis and management. AJR Am J Roentgenol.

[42] Liu F, Zhang Q, Huang C, Shi C, Wang L, Shi N (2020). CT quantification of pneumonia lesions in early days predicts progression to severe illness in a cohort of COVID-19 patients. Theranostics.

[43] Nishiyama A, Kawata N, Yokota H, Sugiura T, Matsumura Y, Higashide T (2020). A predictive factor for patients with acute respiratory distress syndrome: CT Lung volumetry of the well-aerated region as an automated method. Eur J Radiol.

[44] Sumikawa H, Johkoh T, Yamamoto S, Yanagawa M, Inoue A, Honda O (2009). Computed tomography values calculation and volume histogram analysis for various computed tomographic patterns of diffuse lung diseases. J Comput Assist Tomogr.

[45] Bankier AA, Madani A, Gevenois PA (2002). CT quantification of pulmonary emphysema: assessment of lung structure and function. Crit Rev Comput Tomogr.

[46] Alihodzic A, Tuba M (2014). Improved bat algorithm applied to multilevel image thresholding. Sci World J.

[47] Ohkubo H, Nakagawa H, Niimi A (2018). Computer-based quantitative computed tomography image analysis in idiopathic pulmonary fibrosis: a mini review. Respir Investig.

[48] Li K, Fang Y, Li W, Pan C, Qin P, Zhong Y (2020). CT image visual quantitative evaluation and clinical classification of coronavirus disease (COVID-19). Eur Radiol.

[49] Lanza E, Muglia R, Bolengo I, Santonocito OG, Lisi C, Angelotti G (2020). Quantitative chest CT analysis in COVID-19 to predict the need for oxygenation support and intubation. Eur Radiol.

[50] Bressem KK, Adams LC, Albrecht J, Petersen A, Thieß HM, Niehues A (2020). Is lung density associated with severity of COVID-19?. Pol J Radiol.

[51] Salvatore C, Roberta F, Angela L, Cesare P, Alfredo C, Giuliano G (2021). Clinical and laboratory data, radiological structured report findings and quantitative evaluation of lung involvement on baseline chest CT in COVID-19 patients to predict prognosis. Radiol Med.

[52] Romanov AM, Yang S, Fabian C, Franzeck FC, Sommer G (2021). Automated CT lung density analysis of viral pneumonia and healthy lungs using Deep learning-based segmentation, histograms and HU thresholds. Diagnostics.

[53] Rubin GD, Ryerson CJ, Haramati LB, Sverzellati N, Kanne JP, Raoof S (2020). The role of chest imaging in patient management during the COVID-19 pandemic: a multinational consensus statement from the fleischner society. Chest.

[54] Bos LDJ, Paulus F, Vlaar APJ, Beenen LFM, Schultz MJ (2020). Subphenotyping acute respiratory distress syndrome in patients with COVID-19: consequences for ventilator management. Ann Am Thorac Soc.

[55] Gibson PG, Qin L, Puah SH (2020). COVID-19 acute respiratory distress syndrome (ARDS): clinical features and differences from typical pre-COVID-19 ARDS. Med J Aust.

